# Nitro drugs for the treatment of trypanosomatid diseases: past, present, and future prospects

**DOI:** 10.1016/j.pt.2014.04.003

**Published:** 2014-06

**Authors:** Stephen Patterson, Susan Wyllie

**Affiliations:** 1 Division of Biological Chemistry and Drug Discovery, Sir James Black Centre, College of Life Sciences, University of Dundee, Dundee, UK; 2 Division of Biological Chemistry and Drug Discovery, Wellcome Trust Biocentre, College of Life Sciences, University of Dundee, Dundee, UK

**Keywords:** trypanosomatids, nitroaromatics, nitroreductase, pro-drugs, bioactivation

## Abstract

There is an urgent need for new, safer, and effective treatments for the diseases caused by the protozoan parasites *Trypanosoma brucei*, *Trypanosoma cruzi*, and *Leishmania* spp. In the search for more effective drugs to treat these ‘neglected diseases’ researchers have chosen to reassess the therapeutic value of nitroaromatic compounds. Previously avoided in drug discovery programs owing to potential toxicity issues, a nitro drug is now being used successfully as part of a combination therapy for human African trypanosomiasis. We describe here the rehabilitation of nitro drugs for the treatment of trypanosomatid diseases and discuss the future prospects for this compound class.

## Highlights

• Two nitro drugs are currently used in the treatment of trypanosomatid diseases.
• Several new nitroaromatics are being developed against the trypanosomatid diseases.
• Many nitro drugs and drug candidates act as prodrugs which require bioactivation.
• Nitroaromatics can have disparate mechanisms of action in trypanosomatid parasites.

## The urgent need for new drugs for trypanosomatid diseases

The genus Kinetoplastida is responsible for diseases such as human African trypanosomiasis (HAT), Chagas disease (CD), and the leishmaniases (Table 1). Collectively, these ‘neglected’ diseases (see Glossary) cause more than 120 000 fatalities annually and the loss of ∼5 000 000 disability-adjusted life years [1]. Some of the poorest areas of the world are afflicted by these vector-borne parasites, and the accompanying economic burden provides an obstacle to improving human health [2]. Current treatments for these diseases are not ideal, with issues such as unacceptable toxicity [3], acquired drug resistance [4], prolonged hospitalization, and cost [1]. Therefore, there is a compelling need for new treatments.

## Nitroaromatic drugs

Compounds containing a nitroaromatic group (Box 1) are used to treat a wide variety of indications including Parkinson's disease, angina, and insomnia [5–7]. Additionally, several nitroaromatics are used as anti-infective agents, including drugs to treat parasitic infections [8,9]: for example, nitazoxanide is approved for giardiasis and cryptosporidiosis [10]; metronidazole for trichomoniasis, giardiasis, and amoebiasis [11]; and nifurtimox for CD and HAT.

The presence of a nitro group in a compound can result in several toxicity issues including carcinogenicity, hepatotoxicity, mutagenicity, and bone-marrow suppression [12,13]. Consequently, some nitro drugs are avoided where there are suitable alternatives; for example, other antibiotics are preferred to chloramphenicol [14]. In addition, the risk of nitro drug toxicity can be reduced by monitoring for side effects; for instance, liver function is measured in those taking tolcapone [5]. Many nitroaromatics require bioactivation (Box 2) to exert their action. However, unwanted bioactivation can also cause toxic side effects [15]. Thus, the very feature which makes the nitro group indispensable for drug action may also render it unsuitable for administration. Identifying the human enzymes which activate nitro drugs may provide strategies to reduce toxicity. For example, nifurtimox is an alcohol dehydrogenase 2 (ALDH2) substrate, and it has been proposed that coadministration with ALDH2 inhibitors could reduce toxicity [16].

Pharmaceutical companies have increased their use of compound metrics and predictors of drug-likeness in an attempt to reduce failures in the drug development process [17]. One result of this is that compounds containing nitro groups are routinely removed from screening collections because the functionality is classified as a ‘structural alert’ [13,18]. Likewise, medicinal chemists are unlikely to synthesize nitroaromatics during the drug development process. This will reduce the number of nitroaromatics under development and may result in missed opportunities.

## Nitro drugs and the trypanosomatids: an historical perspective

The chemotherapeutic potential of nitroaromatic compounds for HAT was first recognized in the 1950s. The 5-nitrofuran derivative nitrofurazone demonstrated curative properties against experimental *T. brucei gambiense* infections in mice [19,20]. These findings, combined with the knowledge that the drug was both central nervous system penetrant and orally bioavailable, prompted clinical trials of nitrofurazone for the treatment of both *T. b. gambiense* and *T. b. rhodesiense* patients [20–22]. Notably, many of the patients recruited in these trials were refractory to the current therapies using pentamidine, suramin, and melarsoprol. In the absence of alternative therapies for these ‘hopeless’ patients, nitrofurazone elicited cure rates of ∼50%. However, the neuropathological toxicity associated with high doses of nitrofurazone led to the trials being suspended. The efficacy of nitrofurazone was also assessed in animal models of CD [23,24] and in both cutaneous and visceral leishmaniasis (VL) [25]. In all cases, nitrofurazone demonstrated only moderate levels of *in vivo* activity and was not further pursued.

**Figure 1:**
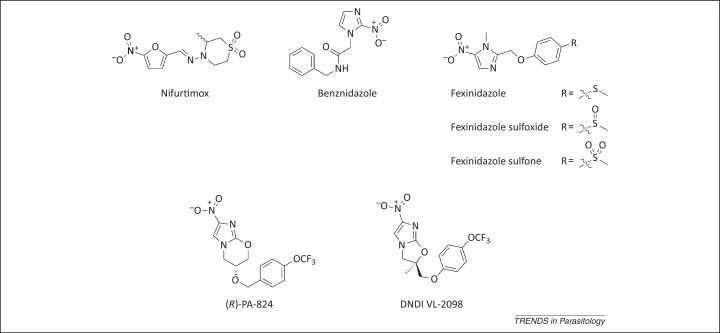
Selected anti-trypanosomatid nitroaromatics and their metabolites. Nitroaromatic compounds used to treat, or are in development for, trypanosomatid diseases. For fexinidazole, both the parent compound and the two metabolites resulting from *in vivo* oxidation are shown.

In the wake of nitrofurazone, several nitroaromatic compounds, including the nitrofuran furaltadone and the nitroimidazole metronidazole [8,19], were found to have anti-trypanosomal activity, but their development was abandoned owing to low efficacy and neurotoxicity. However, continued interest in the therapeutic potential of this compound class led to the discovery of the 5-nitrofuran nifurtimox (Figure 1). Identified by Bayer in *in vitro* screens against *T. cruzi*, nifurtimox was marketed under the brand name Lampit, principally for use in the treatment of acute CD. For many years nifurtimox was considered the front-line therapy for this indication. Nifurtimox is no longer prescribed in Brazil, Chile, Uruguay, or the USA owing to reports of gastrointestinal tract side effects, genotoxicity, low efficacy against particular *T. cruzi* strains, and the emergence of neurotoxicity similar to that encountered during nitrofurazone development.

Nifurtimox has also shown activity against HAT, and the drug is effective against both the acute and chronic stages of *T. b. gambiense* infection. Efficacy was first demonstrated in rat models and then in a limited clinical trial in four European patients [26]. Subsequent trials with various nifurtimox regimens [27,28] revealed that cure rates were variable (30–80%), with toxicity accompanying higher doses and prolonged treatment [29]. Consequently, nifurtimox was not approved as a monotherapy for second-stage HAT and is used primarily to treat patients refractory to existing therapies. Despite the problems associated with its use, nifurtimox has a place in HAT chemotherapy in nifurtimox–eflornithine combination therapy (NECT) [30].

The current drug of choice for acute-stage CD is the 2-nitroimidazole benznidazole (Figure 1). Benznidazole has been used in the treatment of CD for more than 40 years, producing long-term parasitological cure in ∼70% of acute-stage patients. Like nifurtimox, benznidazole is orally administered with treatment consisting of 5–10 mg/kg/day for 30–60 days. Although benznidazole is considered to be tolerated better than nifurtimox, a range of serious side effects are associated with its use, including dermatological reactions [31], agranulocytosis, and polyneuropathy [9]. Owing to these serious adverse effects, recommended drug schedules are frequently not completed, providing ideal conditions for the emergence of drug resistance. In addition to toxicity, Ames assays demonstrate that benznidazole is mutagenic [32].

The value of benznidazole in treating chronic-stage CD is controversial. Although some reports show efficacy in treating chronic infections in mice [33], at present there is insufficient evidence to support routine use of the drug in humans. With this in mind, a multi-centre, double-blind, placebo-controlled trial of benznidazole in patients with chronic Chagas heart disease is underway [34]. Without a viable alternative, hope lies in finding evidence for a beneficial and definitive effect in these patients.

## Reassessing the chemotherapeutic potential of nitroaromatic compounds

Despite the valid safety concerns surrounding nitroaromatic compounds, there is continued interest in their use as therapeutics, particularly for infectious diseases and cancer. In these instances the aim is to develop compounds that are selectively toxic such that the compound kills the infectious agent/cancer cells without harming the host/normal cells [12].

Nitroaromatics can exert cellular toxicity via mechanisms that cannot be replicated by standard ‘drug-like’ molecules (Box 2). Therefore, inclusion of nitroaromatic compounds in screening collections increases the probability of finding a screening hit, especially in cell-based/phenotypic assays. Given the urgent need for new drugs to treat trypanosomatid diseases, and the efficacy of existing nitro drugs for these indications, several laboratories have shown a willingness to identify and develop nitroaromatic anti-trypanosomatid compounds [35–37]. The Drugs for Neglected Diseases initiative (DNDi) has actively chosen to investigate nitroaromatic compounds [38]. Their investigations have proved fruitful, resulting in a trypanosomatid portfolio that contains several nitroaromatics.

Many of the nitroaromatics in development result from drug repurposing programs. For example, the anti-parasitics nitazoxanide and nifurtimox are now in clinical trials for hepatitis C [10] and pediatric neuroblastoma [39], respectively. In a drug repurposing project the compound under investigation has already completed some clinical trials, and therefore the safety and pharmacokinetic properties have been assessed thoroughly [40]. Accordingly, this strategy can both expedite and lower the cost of the drug discovery process. The risk of a project is reduced significantly by using a nitroaromatic proven safe previously; not all nitro drugs are toxic, but it is impossible to be certain that a compound developed *de novo* will be safe. Moreover, the potential cost savings make the repurposing of drugs and clinical candidates against neglected diseases particularly attractive. Indeed, the use of nifurtimox to treat HAT [30] and the development of fexinidazole for VL [41] are examples of repurposing nitroaromatics for trypanosomatid diseases.

## Nitro drugs and the trypanosomatids: drug rediscovery

The combination of eflornithine with nifurtimox was first evaluated for the treatment of late-stage *T. b. gambiense* infection in 2001 as part of a clinical trial comparing three drug combinations: melarsoprol and eflornithine, melarsoprol with nifurtimox, and eflornithine with nifurtimox [42]. The study was aimed at addressing the myriad of disadvantages of existing anti-trypanosomal drugs. The rationale was that using drugs in combination would reduce the likelihood of drug resistance emerging and allow reduced drug doses to be used, thus reducing toxicity without compromising efficacy. Unfortunately, this clinical trial was interrupted on ethical grounds owing to high drug-related mortality reported in the melarsoprol arms of the trial. However, the combination of eflornithine and nifurtimox appeared to be well tolerated and demonstrated sufficiently promising efficacy to warrant further investigation. This drug combination, known as NECT, was assessed in a randomized Phase III non-inferiority trial [30]. The cure rates reported with NECT in this study (96.5%) were comparable to those seen with eflornithine monotherapy, with patients suffering fewer adverse effects. In the combination therapy, intravenous eflornithine is given at 200 mg/kg every 12 h for 7 days, with nifurtimox being administered orally 3 times daily for 10 days. NECT has many advantages over eflornithine monotherapy; specifically the reduced frequency and duration of eflornithine infusions is seen as highly beneficial in terms of cost, logistics, and human resources in areas of poverty. NECT was added to the *Model Lists of Essential Medicines* of the World Health Organization in 2009 and is now being used to treat more than 60% of cases of late-stage *T. b. gambiense* HAT (http://www.doctorswithoutborders.org).

Around the time of the development of NECT, the nitroimidazole megazol also demonstrated promising anti-trypanosomal activity. A megazol–suramin combination was curative in stage 2 HAT rodent models [43,44]. Although subsequently abandoned owing to genotoxicity [45], the perceived potential of megazol at this time, alongside the success of nifurtimox in NECT, encouraged DNDi to initiate a comprehensive search for nitroaromatics with anti-trypanosomatid activity. As a result, 700 selected compounds were assayed for anti-parasitic activity. Among the hit compounds was the 5-nitroimidazole fexinidazole (Figure 1), first described in 1978 [46]. In its first incarnation, fexinidazole demonstrated potent *in vitro* and *in vivo* activity against trichomonads, *Entamoeba histolytica*, *T. cruzi*, and *T. brucei* [47]. When coadministered with suramin, fexinidazole was found to effectively cure the stage 2 mouse model of HAT. However, the clinical development of this drug was not pursued, largely due to the ingrained prejudice against nitroaromatic compounds at that time. Reassessment of the pharmacological and toxicological profile of fexinidazole established the compound as a promising candidate for both the acute and chronic stages of HAT [38]. Significantly, a barrage of assays to detect toxicity, carried out both *in vitro* and *in vivo* assays, confirmed that fexinidazole is non-genotoxic to mammalian cells. In 2009, fexinidazole became the first new trypanosomatid disease clinical candidate for three decades, and is currently undergoing Phase III trials. It is hoped that this nitroimidazole will become the first orally available drug for both stages of HAT.

To date, nitroaromatics have not been widely used in the treatment of the leishmaniases. However, in light of the promise shown by fexinidazole for HAT, the compound was assessed for its potential to treat VL [41]. Winkelmann and Raether had hypothesized that fexinidazole could be metabolized *in vivo* to the sulfoxide and/or sulfone (Figure 1) [46]. Subsequent pharmacokinetic profiling of blood from mice orally dosed with fexinidazole confirmed that this was the case [41]. Indeed, the metabolism is rapid, and the sulfoxide and sulfone blood concentrations exceed that of fexinidazole, strongly suggesting that the metabolites are the therapeutically relevant species *in vivo*. Interestingly, both metabolites of fexinidazole were active against *L. donovani* amastigotes grown in macrophages, whereas the parent compound was inactive [41]. The reasons for this discrepancy in activity are not fully understood. Fexinidazole was subsequently found to be a potent inhibitor of *Leishmania* infection *in vivo*. Administration of a once-daily oral regimen of fexinidazole (200 mg/kg) over 5 days suppressed infection in a mouse model of VL by approximately 98%, a potency comparable to that seen with the current frontline anti-leishmanial miltefosine. In light of these findings, patients are being recruited for a Phase II proof-of-concept study to evaluate the suitability of fexinidazole as a treatment for VL in Sudan (http://www.dndi.org). The versatility of fexinidazole has been further demonstrated by its efficacy as an effective oral treatment of acute and chronic experimental CD caused by benznidazole-susceptible, partially resistant, and resistant *T. cruzi* [48]. This raises the possibility that fexinidazole could act as a panacea for all three trypanosomatid-related diseases.

## Nitro drugs and the trypanosomatids: in the pipeline

Encouraged by the success of NECT and the undoubted potential of fexinidazole, there is now a willingness to investigate further the chemotherapeutic potential of nitro drugs for trypanosomatid diseases. Many of the toxic effects observed after the administration of nitro drugs in the past have resulted from prolonged treatment. The Target Product Profiles (TPP, Table 2) developed for all three trypanosomatid diseases have now stipulated that treatment regimens should be of short duration. With this in mind, researchers and clinicians are now more open to the use of this compound class for the treatment of trypanosomatid diseases.

Several recent reports have described nitroaromatics with promising anti-trypanosomatid activity. For instance, a series of 5-nitro-2-furancarboxylamides showed potent cidal activity against *T. brucei in vitro*. Significantly, the most potent of these compounds was ∼1000-fold more potent than nifurtimox against bloodstream-form parasites [35]. Similarly, a subset of aziridinyl-2-4-dintrobenzyl compounds possessing a 5-amide substituent were identified with cidal activity against *L. donovani* intracellular amastigotes (EC_50_ <100 nM) [37]. The (*R*)-enantiomer of the novel nitroimidazopyran agent, (*S*)-PA-824, currently in Phase II clinical trials for tuberculosis, is a potent inhibitor of *L. donovani*, both *in vitro* and *in vivo.* In the murine model of VL, (*R*)-PA-824 administered orally at 100 mg/kg twice daily resulted in a virtual cure, suppressing infection by >99% [49]. Furthermore, an additional nitroimidazole, DNDI-VL-2098, is at an advanced stage of preclinical development for use in the treatment of VL (http://www.dndi.org). Collectively, these findings suggest that nitroaromatics may play a significant role in the future treatment of these diseases.

## Nitroaromatic compounds: mechanism of action

Given the prominence of nitroaromatics in the drug discovery pipelines of several diseases, concerted efforts are now being made to elucidate their mechanisms of action. As for any small-molecule inhibitor, nitroaromatic compounds can exert their biological effects by binding to one or more molecular targets (usually proteins), and as a result produce the phenotype of interest, which is cell death in the case of anti-parasitics. If this is the case, the nitro group acts like any other functional group by contributing directly or indirectly to one or more target–ligand binding interactions. Moreover, the fact that nitroaromatics can be enzymatically reduced (Box 2) allows them to exert cellular toxicity via additional mechanisms, as demonstrated for the nitro drugs used to treat trypanosomatid diseases [50,51].

Several early studies suggested that trypanosomes bioactivate nifurtimox by a one-electron, type II nitroreductase (NTR)-dependent mechanism, and that the resulting oxidative stress was responsible for the observed cytotoxicity (reviewed in [9]). However, it is now apparent that bioactivation of nifurtimox, benznidazole, and fexinidazole is mediated by type I NTR enzymes (Box 2) [37,41,52]. These recent findings supersede previous studies implicating other enzymes such as trypanothione reductase in the activation of nitroaromatics (reviewed in [9]). Trypanosomatids are unusual in that they possess a bacterial-like type I NTR [52] for which there is no mammalian homolog. Therefore, the selective toxicity observed for these inhibitors may be due to the presence of a specific parasitic bioactivating enzyme which is absent from the host. Unsurprisingly, nitroaromatics selectively activated by the bacterial-like NTR are also Ames positive [32]. This brings into question the suitability of this assay for assessing the mutagenic potential of this compound class within a mammalian host. By recombinantly expressing the catalytic domains of trypanosomatid NTRs, a detailed examination of the mechanism of nitro drug bioactivation is now possible [50,51].

**Figure 2:**
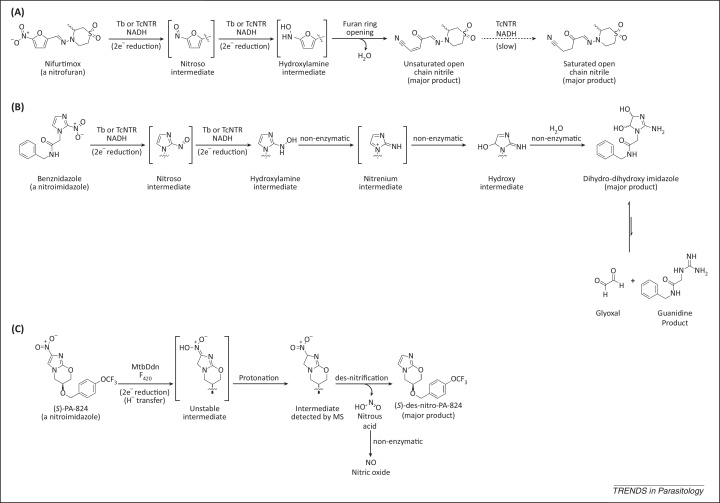
Mechanism of nitroreductase (NTR)-mediated bioactivation of nitroaromatic compounds. **(A)** Nifurtimox undergoes two sequential NTR-catalyzed reductions, followed by ring opening to give a toxic unsaturated open-chain nitrile. Tb, *Trypanosoma brucei*; Tc, *Trypanosoma cruzi*. **(B)** The nitro group of benznidazole is reduced to the hydroxylamine by NTR. Subsequently, a series of non-enzymatic transformations leads to a dihydro-dihydroxy product, which can release glyoxal. **(C)** Unusually, MtbDdn [deazaflavin (F_420_)-dependent nitroreductase (Ddn) of *Mycobacterium tuberculosis* (Mtb)] does not reduce the nitro group of (*S*)-PA-824. Instead, the first step of the bioactivation involves reduction of the C2–C3 bond of the bicyclic imidazooxazine ring system. The unstable product of this reduction is protonated and then undergoes a des-nitrification reaction to give des-nitro-PA-824, with the concomitant production of nitrous acid. Some structures have been abbreviated for clarity. Compounds in square brackets are not experimentally detected. Adapted from [9,61].

Incubation of nifurtimox with *T. brucei* (Tb)NTR or *T. cruzi* (Tc)NTR leads to the formation of an unsaturated open-chain nitrile product (Figure 2A) [50]. No intermediate metabolites were observed in this transformation. However, it can be inferred that the nitro group has undergone two sequential two-electron reductions, giving first a nitroso and then a hydroxylamine intermediate. Subsequent furan ring-opening with loss of water would lead to the observed unsaturated open-chain nitrile. TcNTR reduces the carbon–carbon double bond of this product metabolite to give a saturated open-chain nitrile, but only following prolonged incubation. Therefore, this additional reduction is unlikely to be physiologically relevant and is hence not involved in the drug mode of action. The unsaturated open-chain nitrile is also toxic against bloodstream-form *T. brucei* parasites *in vitro*, with a potency similar to that measured for nifurtimox (EC_50_ values of 5.3 μM and 2.9 μM, respectively [50]); thus the NTR-dependant production of the unsaturated open-chain nitrile could possibly be responsible for cell death following treatment with nifurtimox. Interestingly, the unsaturated open-chain nitrile is equipotent against *T. brucei* and mammalian cells *in vitro*, whereas nifurtimox itself is 10-fold more potent against *T. brucei*, consistent with the paradigm that drug selectivity is achieved through selective bioactivation by the parasite. The mechanism by which the unsaturated open-chain nitrile causes cell death is undetermined. However, the unsaturated open-chain nitrile contains a Michael acceptor, a chemical moiety that is known to react with sulfhydryl groups in proteins or other biomolecules [53]. Therefore, it can be hypothesized that the unsaturated open-chain nitrile irreversibly inhibits one or more essential proteins by covalently binding to an accessible and/or activated cysteine residue(s), leading to cell death.

The mechanisms underlying resistance to nifurtimox in *T. brucei* were recently assessed using a genome-scale RNA interference target sequencing (RITseq) screen [54]. In this elegant study, eight genes were identified as being strongly associated with nifurtimox resistance. Of these eight genes, six directly or indirectly reaffirmed the dominant role of the NTR in nifurtimox activation.

Benznidazole also serves as a substrate for TcNTR and TbNTR [51]. As for nifurtimox, the nitro group undergoes two sequential two-electron reductions to give a hydroxylamine intermediate (Figure 2B), which was detected by liquid chromatography–mass spectrometry (LCMS) following incubation with recombinant NTR and NADH. Unlike nifurtimox, the reduced benznidazole does not undergo a ring-opening reaction. Instead, a series of non-enzymatic transformations convert the hydroxylamine intermediate into a 4,5-dihydro-4,5-dihydroxyimidazole (Figure 2B) [55,56]. Note, both the *cis* and *trans* isomers of this diol were detected by LCMS analysis of the reaction mixture [51]. In aqueous solution these dihydro-dihydroxyimidazoles are known to exist in equilibrium with glyoxal and a substituted guanidine product (Figure 2B) [57]. Glyoxal is a highly toxic, reactive dialdehyde which is capable of chemically modifying proteins, lipids, and nucleotides [58]. Therefore, it was hypothesized that the formation of glyoxal is responsible in part for the anti-trypanosomal action of benznidazole. In support, addition of guanosine to the reaction mixture leads to the formation of a guanosine–glyoxal adduct [51]. However, the production of glyoxal from dihydro-dihydroxyimidazoles is extremely slow [57], meaning that glyoxal production is unlikely to be the sole cytotoxic mechanism.

Laboratory-generated nifurtimox-resistant *T. brucei* are cross-resistant to fexinidazole *in vitro* and *in vivo* [59], suggestive of a common mechanism of action for these two compounds. Additionally, transgenic *L. donovani* parasites overexpressing *L. major* NTR are approximately 20-fold more sensitive to fexinidazole *in vitro* [41,60]. These observations are consistent with the hypothesis that the bioactivation of fexinidazole is dependent upon NTR enzymes in trypanosomatids. However, it is currently unknown which fexinidazole reduction product(s) is responsible for the cidal activity of the compound.

Under anaerobic conditions the anti-mycobacterial action of (*S*)-PA-824 is dependent upon reductase-mediated bioactivation [61]. This reduction is catalyzed by an unusual deazaflavin (F_420_)-dependent nitroreductase (Ddn) [61,62]. Incubation of (*S*)-PA-824 with recombinant *Mycobacterium tuberculosis* (Mtb)Ddn leads to the formation of multiple products, the most abundant being (*S*)-des-nitro-PA-824 (Figure 2C) [61,63]. This bioactivation also produces nitric oxide. Transcriptional profiling suggests that respiratory poisoning by nitric oxide is responsible for the anti-mycobacterial action of (*S*)-PA-824 [64]. The *L. donovani* genome does not contain a MtbDdn homolog. Therefore, it was hypothesized that the type I NTR might instead be responsible for bioactivation of (*S*)- and (*R*)-PA-824 in *Leishmania*. Transgenic *L. donovani* promastigotes overexpressing NTR do not show increased sensitivity to (*S*)-, or (*R*)-PA-824 [49], and a close analog of PA-824 is not a substrate for TbNTR [51]. These results demonstrate that (*S*)- and (*R*)-PA-824 are not activated by the trypanosomatid type I NTR. Both (*R*)- and (*S*)-des-nitro-PA-824 are inactive against *L. donovani in vitro* [49], confirming that the nitro group is important for the anti-leishmanial activity of PA-824. If the mechanism of action of PA-824 does involve bioactivation, then this putative reduction is mediated by an as-yet unidentified enzyme(s). Under aerobic conditions the anti-mycobacterial action of (*S*)-PA-824 involves inhibition of mycolic acid biosynthesis [65]. *L. donovani* do not possess the mycolic acid biosynthetic pathway, meaning that this mechanism cannot be implicated in the anti-leishmanial activity of PA-824.

## Nitro drugs: implications of widespread use

The simultaneous development of multiple nitroaromatics for the trypanosomatid diseases potentially has serious implications. It is considered undesirable to have several compounds with a shared mode of action under development for a single indication. Such a scenario leaves the pipeline vulnerable to multiple compound failures associated with a single mechanism. Specifically, parasites resistant to one nitroaromatic can be cross-resistant to a second; for example, nifurtimox-resistant *T. brucei* are cross-resistant to fexinidazole [59]. In this case resistance is largely due to their common NTR-mediated mode of action. This brings into question the rationale of developing further NTR-activated nitroaromatic compounds for the treatment of trypanosomatid-related diseases. Therefore, it is important to determine if new anti-trypanosomatid nitroaromatics are NTR-activated early in development. The primary physiological function of trypanosomatid NTRs is not to reduce exogenous nitroaromatics. NTR is essential in *Leishmania* and *T. brucei*, and is required for virulence in *T. cruzi* [52,60,66]. RITseq data suggest that this essential role is linked to ubiquinone biosynthesis [54]. The essentiality of NTR has implications for drug resistance, and suggests that NTR is itself a potential drug target.

The target product profile (TTP) for VL (Table 2) stipulates that newly developed drugs should be suitable for combination therapy. Clearly, the partner drugs in any combination therapy must have distinct modes of action. Despite both being nitroimidazoles, (*R*)-PA-824 and fexinidazole do not share a common mechanism of action [49]. Therefore, a single mutation is unlikely to confer resistance to both compounds. In combination, fexinidazole and (*R*)-PA-824 have an additive effect against *L. donovani in vitro*. This raises the possibility that these two nitroaromatics could be co-developed as a VL oral combination therapy.

## Concluding remarks and future perspectives

The launch of NECT in 2009 not only heralded the first new therapy for HAT in over three decades, but also demonstrated that nitro drugs could be used to treat trypanosomatid diseases safely and effectively. As a result there has been a marked increase in the number of nitroaromatic compounds in development for these diseases, including for the first time a clinical candidate for VL. During the same period our understanding of the anti-trypanosomal mechanisms of action of nitro drugs has substantially improved.

By using recombinant proteins it has been possible to characterize the bioreduction of nifurtimox and benznidazole at the molecular level. Our understanding is incomplete, and the anti-leishmanial mechanism(s) of action of (*R*)-PA-824 and DNDI-VL-2098 remain to be determined. Defining the mechanism(s) of action of these compounds should be a priority. A more complete understanding of how these nitroimidazoles kill parasites will inform drug combination strategies, and may identify novel drug targets/mechanisms of cytotoxicity which could be exploited by *de novo* target-based drug discovery.

Recent research has revealed the potential of nitroaromatics to treat neglected diseases and has eroded the long-held prejudice against them. Mindful of the risks associated with this compound class, future drug development should proceed with caution rather than disregarding nitroaromatics out of hand.

## Figures and Tables

**Figure I:**
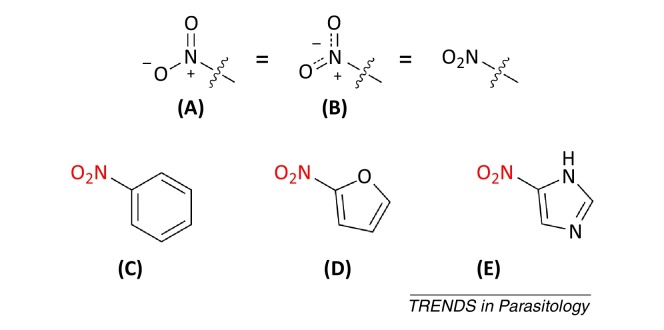
Alternative nitro group representations and examples of nitroaromatics.

**Figure I:**
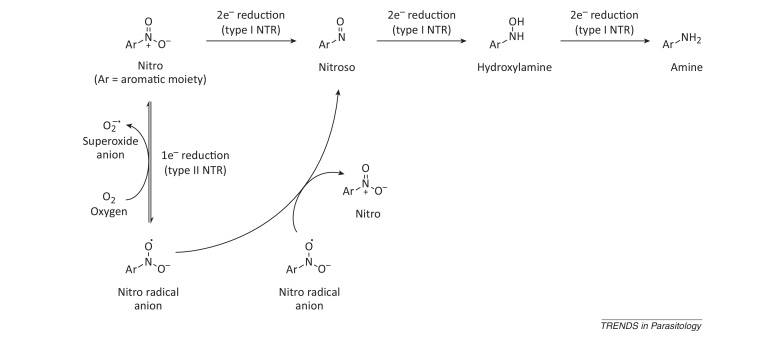
One- and two-electron reduction of nitroaromatics.

**Table 1:** Trypanosomatid diseases

Disease	Human African trypanosomiasis (HAT)	Chagas disease	Visceral leishmaniasis (VL)
Causative agents	*Trypanosoma brucei gambiense* *Trypanosoma brucei rhodesiense*	*Trypanosoma cruzi*	*Leishmania donovani* *Leishmania infantum*
Areas of endemicity	West and central Africa (*T. b. gambiense*) East and southern Africa (*T. b. rhodesiense*)	Central and South America	India, Bangladesh, Nepal, Sudan, Ethiopia, and Brazil
Deaths per annum	∼30 000 [1]	∼10 000 [67]	∼50 000 [68]
Pathology and symptoms	*Early stage:* parasites proliferate in the blood and lymphatic systems. This results in patients generally feeling unwell, experiencing indeterminate symptoms such as headaches *Late stage:* parasite invasion of the central nervous system results in progressive neurological breakdown. Fatal if untreated	*Acute:* a general feeling of being unwell accompanies the acute phase following infection. *Indeterminate stage/chronic:* a long, asymptomatic, indeterminate phase can follow. Inflammatory responses to residual parasites can induce progressive and sometimes fatal inflammatory damage to the heart, esophagus, colon, or other organs [69]	Initial skin lesions at the site of infection. 2–8 months following infection migration of parasites to the liver and the spleen results in gross inflammatory reactions within the viscera. Fatal if untreated [69]
Current front-line therapies	*Early stage:* pentamidine and suramin *Late stage:* melarsoprol, eflornithine monotherapy^a^ and NECT (nifurtimox-eflornithine combination therapy)^a^	*Acute:* nifurtimox and benznidazole *Indeterminate and chronic stages:* no standard treatments	Amphotericin B and lipid formulations Miltefosine Pentavalent antimonials Paromomycin

a Not effective against *T. b. rhodesiense* infection.

**Table 2:** Ideal target product profiles (TPP) for trypanosomatid diseases^a^

Human African trypanosomiasis^b^	Visceral leishmaniasis^c^	Chagas disease^c^
Active against all subspecies	Active against all species	Active against all strains
Active against melarsoprol-refractory strains	Active against resistant strains	Active against nitrofuran- and nitroimidazole-resistant *T. cruzi* strains
Efficacy against early- and late-stage disease	Compatible for combination therapy	Superior to benznidazole and effective for both chronic and late-stage disease
Oral formulation	Oral formulation	Oral formulation
Curative in 14 days (late stage)	Once daily oral treatment (10 days)	Once daily oral treatment (30 days)
Cheaper than current treatments	<$10/course	No cost defined
Safe in pregnancy	Safe in pregnancy and in immunocompromised patients	No genotoxicity; no teratogenicity; no negative inotropic effect; significant pro-arrhythmic potential
Stable in tropical conditions	Stable in tropical conditions	Stable in tropical conditions

a As an aid to future drug discovery, key features desired from any drug to be used in the treatment each of the neglected diseases have now been collated and defined as TPPs.

b Adapted from [75].

c Adapted from http://www.dndi.org.

## Box 1 What is a nitroaromatic compound?

A nitro group is a chemical functional group containing a nitrogen atom bound to two oxygen atoms and one carbon atom which connects the group to the rest of the molecule. The nitrogen atom in a nitro group is positively charged, and the two oxygen atoms share a negative charge. A nitro group can be drawn in several ways; commonly the whole group is abbreviated as NO_2_. Alternatively, the nitro group can be represented by the structures **(A)** or **(B)** in Figure I.

A nitroaromatic compound is a molecule in which one or more nitro groups are directly attached to an aromatic ring system. For example, nitrobenzene **(C)**, 2-nitrofuran **(D)**, and 5-nitroimidazole **(E)** in Figure I.

Chemically, the nitro group possesses a unique combination of properties; it is strongly electron-withdrawing, small, polar, and can form hydrogen bonds [70]. In addition, the nitro group can be bioactivated by enzymatic reduction to give reactive species (Box 2, Figure 2). In many cases these reactive species are responsible for the biological effects of nitro drugs [15], in which case the nitroaromatic compound is acting as a pro-drug.

## Box 2 Reduction of nitro groups by NTR enzymes

In biological systems, nitro groups can undergo enzymatic reduction, which can proceed by either a one or two electron mechanism [71]. Nitro group reduction can be catalyzed by NTR enzymes, of which there are two classes. Type I (oxygen-insensitive) NTRs perform two-electron reductions, and type II (oxygen-sensitive) NTRs perform one-electron reductions [72] (Figure I).

Sequential two-electron reduction of nitro groups produces amines via nitroso and hydroxylamine intermediates. The nitroaromatics and amines are relatively stable. However, the nitroso and hydroxylamine intermediates can react with biomolecules to exert toxic and mutagenic effects [15,73]. In addition, there is evidence to suggest that hydroxylamines are converted to reactive nitrenium ions, which can react with DNA [73].

One-electron reduction of a nitro group produces a nitro radical anion. This anion is not stable, and under aerobic conditions is reoxidized back to a nitro group by molecular oxygen, which is in turn reduced to form a reactive superoxide anion. This process is termed the ‘futile cycle’, and in some cases is responsible for the toxic effects of nitro compounds. In the absence of oxygen, two nitro radical anions can undergo a disproportionation reaction to form one molecule of the parent nitroaromatic and a nitroso compound. It has been proposed that this process may produce nitroso compounds in biological systems [74].

## Glossary

Ames test: a widely employed *Salmonella*-based cell assay used in medicinal chemistry projects to determine whether a given compound is mutagenic.
Deazaflavin (F_420_): an unusual 8-hydroxy-5-deazaflavin cofactor closely related to FMN.
Deazaflavin-dependent nitroreductase (Ddn): a nitroreductase from *Mycobacterium tuberculosis* which catalyzes the deazaflavin-dependent reduction of nitroimidazoles such as PA-824.
Drugs for Neglected Diseases Initiative (DNDi): an independent, not-for-profit drug research and development organization. DNDi research focuses on the area of neglected diseases, in particular on leishmaniasis, human African trypanosomiasis, and Chagas disease (http://www.dndi.org).
Drug-like: a small molecule which falls within a predefined range of physicochemical parameters. The parameters differ slightly depending upon the indication and the desired mode of administration. The most commonly applied parameters for an orally available drug are: molecular weight <500 Daltons, partition coefficient log *P* <5, ≤5 hydrogen bond donors, and ≤10 hydrogen bond acceptors. In addition, particular structural motifs are commonly excluded.
Drug repurposing: investigating the effectiveness of an existing drug for a new indication.
Effective concentration 50 (EC_50_): the concentration of drug required to kill 50% of parasites in an *in vitro* assay.
Flavin mononucleotide (FMN): a cofactor in many oxidoreductase reactions.
Liquid chromatography–mass spectrometry (LCMS): an analytical technique wherein the components of a mixture are separated by high performance liquid chromatography (HPLC) coupled to a mass spectrometer (and usually a UV detector).
Neglected disease: a disease for which there is a disproportionately low level of investment and research into the development of treatments relative to the size of the affected population. Neglected diseases typically affect developing countries, and the at-risk populations have insufficient purchasing power to allow the profitable development of therapeutics.
Nitroreductase (NTR) enzymes: a family of FMN- or FAD-dependent enzymes capable of metabolizing nitroaromatic compounds. NTRs utilize NADH or NADPH as a reductive cofactor. The NTRs are subdivided into two classes depending upon their reaction mechanism.
Pro-drug: a compound that is administered in an inactive/less than fully active form that requires conversion to its active form through a normal metabolic process.
Small molecule: a low molecular weight compound (usually below 500 Daltons). The term is usually used to distinguish small chemical compounds from larger biomolecules – for example, peptides.
Structural alert: a medicinal chemistry term used to describe a chemical moiety, or functional group, that is known to impart undesirable properties to a molecule – for example, a group known to be reactive or unstable *in vivo*.
Type I nitroreductases: FMN-dependent NTRs that catalyze the reduction of nitroaromatic compounds by a two-electron mechanism. Type I NTRs are oxygen-insensitive, being able to carry out reactions under both aerobic and anaerobic conditions.
Type II nitroreductases: NTRs that catalyze the reduction of nitroaromatic compounds by a one-electron mechanism. In the presence of oxygen, this reduction leads to the production of superoxide anions.
